# The Transcriptional Cofactor Nab2 Is Induced by TGF-β and Suppresses Fibroblast Activation: Physiological Roles and Impaired Expression in Scleroderma

**DOI:** 10.1371/journal.pone.0007620

**Published:** 2009-10-26

**Authors:** Swati Bhattacharyya, Jun Wei, Denisa S. Melichian, Jeffrey Milbrandt, Kazuhiko Takehara, John Varga

**Affiliations:** 1 Division of Rheumatology, Northwestern University Feinberg School of Medicine, Chicago, Illinois, United States of America; 2 Division of Neurology, Washington University School of Medicine, St. Louis, Missouri, United States of America; 3 Department of Dermatology, Kanazawa University, Kanazawa, Japan; Tufts University, United States of America

## Abstract

By stimulating collagen synthesis and myofibroblasts differentiation, transforming growth factor-β (TGF- β) plays a pivotal role in tissue repair and fibrosis. The early growth response-1 (Egr-1) transcription factor mediates profibrotic TGF-β responses, and its expression is elevated in biopsies from patients with scleroderma. NGF1-A-binding protein 2 (Nab2) is a conserved transcriptional cofactor that directly binds to Egr-1 and positively or negatively modulates Egr-1 target gene transcription. Despite the recognized importance of Nab2 in governing the intensity of Egr-1-dependent responses, the regulation and function of Nab2 in the context of fibrotic TGF-β signaling is unknown. Here we show that TGF-β caused a time-dependent stimulation of Nab2 protein and mRNA in normal fibroblasts. Ectopic expression of Nab2 in these cells blocked Egr-1-dependent transcriptional responses, and abrogated TGF-β-induced stimulation of collagen synthesis and myofibroblasts differentiation. These inhibitory effects of Nab2 involved recruitment of the NuRD chromatin remodeling complex to the COL1A2 promoter and were accompanied by reduced histone H4 acetylation. Mice with targeted deletion of Nab2 displayed increased collagen accumulation in the dermis, and genetic or siRNA-mediated loss of Nab2 in fibroblasts was associated with constitutively elevated collagen synthesis and accentuation of Egr-1-dependent TGF-β responses in vitro. Expression of Nab2 was markedly up-regulated in skin biopsies from patients with scleroderma, and was localized primarily to epidermal keratinocytes. In contrast, little Nab2 could be detected in dermal fibroblasts. These results identify Nab2 as a novel endogenous negative regulator of Egr-1-dependent TGF-β signaling responsible for setting the intensity of fibrotic responses. Defective Nab2 expression or function in dermal fibroblasts might play a role in persistent fibrotic responses in scleroderma.

## Introduction

Scleroderma is characterized by autoimmunity, vascular injury and tissue fibrosis [1], [2]. Fibroblast activation resulting in collagen overproduction and myofibroblasts differentiation plays a central role in the development and progression of tissue fibrosis in the skin and internal organs. The multifunctional cytokine transforming growth factor-β (TGF-β) is a potent stimulus for fibroblast activation, and is strongly implicated in the pathogenesis of scleroderma [3], [4]. While both the canonical Smad pathway and Smad-independent signaling cascades have been shown to mediate intracellular TGF- β signaling, the molecular mechanisms regulating fibrotic TGF-β responses remain incompletely understood [5]. Identification and functional characterization of the transcription factors and cofactors that mediate TGF-β responses has significant implications for the development of anti-fibrotic therapies [6].

The early growth response family of zinc finger transcription factors includes Egr-1 (also known as NGFI-A), Egr-2, Egr-3 and Egr-4 [7], [8], [9]. Members of the Egr-1 family are implicated in the regulation of cell growth, differentiation and apoptosis. Although most normal cells show negligible basal expression of Egr-1, synthesis is induced in a rapid and generally transient manner by a variety of extracellular signals generated during stress and injury. Stimuli for Egr-1 expression include growth factors, hypoxia, reactive oxygen species, ultraviolet light and mechanical injury. We demonstrated previously that TGF-β was an additional stimulus for inducing Egr-1 expression in normal fibroblasts [10]. The response involved a Smad-independent signal transduction pathway with sequential activation of MEK1, ERK1/2 and Elk-1, as well as the non-receptor protein kinase c-Abl [11], [12]. Ectopic Egr-1 was sufficient for stimulating collagen gene expression in the absence of TGF-β. We demonstrated that levels of Egr-1 were markedly elevated in mice with bleomycin-induced scleroderma (10). Moreover, the expression of Egr-1 was found to be enhanced in skin and lung biopsies from patients with scleroderma [10], [11]. Together, these observations point to a hitherto unrecognized fundamental role for Egr-1 in the pathogenesis of fibrosis in mouse and man.

Under normal conditions, Egr-1 expression and activity are tightly regulated. One of the factors implicated in regulation of Egr-1 is NGF1-A-binding protein 2 (Nab2), a 55 kD nuclear protein originally identified based on its ability to interact with Egr-1 and inhibit its transcriptional activity [13], [14]. Subsequent studies revealed that Nab2 lacks DNA-binding activity, but can positively or negatively modulate the expression of Egr-1 target genes via direct interaction with Egr-1 [15]. Nab2 shares conserved N-terminal NCD1 (Egr-1 interaction) and C-terminal NCD2 (transcriptional regulation) domains with another Egr-1 binding protein called Nab1. However, while Nab2 is an inducible modulator of transcription, Nab1 is constitutively expressed in most tissues, and functions as general transcriptional regulator [16]. The synthesis of Nab2 is stimulated by some of the same signals that also induce Egr-1, suggesting that Nab2 might function in a negative feedback for Egr-1 activity [14]. Although Nab2 has been implicated in macrophage development, cardiac hypertrophy, peripheral neuropathy and prostate cancer, to date its in vivo functions are poorly understood. Mice with targeted deletion of Nab2 showed no obvious phenotype [17]. In contrast, mice lacking both Nab1 and Nab2 showed profound hypomyelination and died at an early age [17].

Despite the potentially important role of Nab2 in modulating Egr-1 activity and target gene expression, its regulation remains incompletely understood. Furthermore, the expression and function of Nab2 in the context of physiologic and pathological fibrogenesis are unknown. Here we show that TGF-β stimulates the expression of Nab2 in normal skin and lung fibroblasts. The Nab2 response is slightly delayed compared to Egr-1, and involves a Smad-independent MAP kinase intracellular signaling cascade. Ectopic Nab2 blocked Egr-1-dependent transcriptional responses, and prevented the stimulation of collagen synthesis and myofibroblasts differentiation. These inhibitory effects of Nab2 were accompanied by recruitment of the histone deacetylase HDAC1 and attenuated histone H4 hyperacetylation at the COL1A2 promoter. Mice null for Nab2 showed increased accumulation of collagen in the skin. Genetic or siRNA-mediated repression of Nab2 in fibroblasts was associated with constitutive Egr-1 activity, increased collagen synthesis, and enhancement of Egr-1-dependent TGF-β responses in vitro. Skin biopsies from patients with scleroderma were characterized by markedly elevated Nab2 expression, localized primarily in the nuclei of epidermal keratinocytes and cells lining dermal appendages. In contrast, fibroblasts in the lesional skin had only scant Nab2 expression. These results demonstrate, for the first time, that Nab2 functions as an inducible inhibitor of Egr-1-mediated TGF-β signaling, and has an important physiologic role in modulating the intensity or duration of fibrotic responses. Impaired Nab2 expression or function might contribute directly to the development or progression of fibrosis in scleroderma.

## Materials and Methods

### Cell culture and reagents

Primary cultures of dermal fibroblasts were established by explantation from neonatal foreskin, and studied at early (<8) passage [18]. Mouse NIH3T3 fibroblasts were obtained from the American Type Culture Collection (Mansassas, VA). Fibroblasts were established from Nab2^−/−^ mice and wildtype littermate embryos and studied in parallel. Fibroblast cultures were maintained in modified Eagle's medium (EMEM) or Dulbecco's modified Eagle's medium (DMEM) supplemented with 10% fetal calf serum (FCS) (Gibco BRL, Grand Island, NY), 1% vitamin solutions, and 2 mM L-glutamine. Normal human lung fibroblasts (NHLF) were obtained from Clonetics (CC-2512, Cambrex, Walkersville, MD) and maintained in FGM®-2 media (CC-3132) (Cambrex). All other tissue culture reagents were from Biowhittaker (Walkersville, MD). For experiments, cultures were placed in fresh serum-free media containing 0.1% bovine serum albumin (BSA) for 24 h prior to addition of indicated concentrations of TGF-β1 (PeproTech, Rocky Hill, NJ).

### Real-time quantitative PCR

At the end of the experiments, total RNA was isolated from fibroblasts and reverse-transcribed to cDNA using Reverse Transcription System (Promega, Madison, WI) [11]. The products (50 ng) were amplified using SYBR Green PCR Master Mix (Applied Biosytems, Foster City, CA) with primers (shown in Table 1) on the Applied Biosystems 7500 Prism Sequence Detection System. Tissue mRNA levels were determined by real-time quantitative PCR [19]. Total RNA was isolated from skin tissue using Trizol Reagent (Invitrogen, Carlsbad, CA), purified and reverse transcribed, followed by real-time qPCR.

**Table 1:** Oligonucleotides used for Real-Time Quantitative PCR.

**Human**	
Egr-1	Forward: 5′-TGCGGCAGAAGGACAAGAAAGC-3′
	Reverse: 5′-TGAGGAAGGGAAGCTGCTGACC-3′
COL1A1	Forward: 5′- CCAGAAGAACTGGTACATCAGCA-3′
	Reverse: 5′- CGCCATACTCGAACTGGGAAT-5′
COL1A2	Forward: 5′- GATGTTGAACTTGTTGCTGAGG-3′
	Reverse: 5′- TCTTTCCCCATTCATTTGTCTT-3′
CTGF	Forward: 5′- TTGCGAAGCTGACCTGGAAGAGAA-3′
	Reverse: 5′- AGCTCGGTATGTCTTCATGCTGGT-3′
Nab2	Forward: 5′-TGACAGCCAGAAGGAAGAGGA-3′
	Reverse: 5′-AGGTGCTCTCTCTCGGGCTACTT-3′
Actin	Forward: 5′-AATGTCGCGGAGGACTTTGAT-3′
	Reverse: 5′-AGGATGGCAAGGGACTTCCTG-3′
**Mouse**	
COL1A1	Forward: 5′- CCTGAGTCAGCAGATTGAGAA-3′
	Reverse:5′ACTGAACTTGACCGTACACCAGTACTCTCCGCTCTTCAA-3′
COL1A2	Forward: 5′-CCGTGCTTCTCAGAACATCA-3′
	Reverse: 5′-CTTGCCCCATTCATTTGTCT-3′
GAPDH	Forward: 5′-GTCGTGGATCTGACGTGCC-3′
	Reverse: 3′-GATGCCTGCTT CACCACCTT-3′
Egr-1	Forward: 5′-TTTGCCTCCGTTCCACCTGC-3′
	Reverse: 5′-TGCCAACTTGATGGTCAT GCGC-3′
Nab2	Forward: 5′-GAGGAGGGGTTGCTGGACCG-3′
	Reverse: 5′- GGCTGGAGGCAAAGTCCG-3′
αSMA	Forward: 5′-CAGCGGGCATCCACGAA-3′
	Reverse: 5′-GCCACCGATCCAGACAGA-3′
18s rRNA	Forward: 5′-TTCGAACGTCTGCCCTATCA-3′
	Reverse: 5′-ATGGTAGGCACGGCGACTA -3′

### Western Analysis

At the end of the experiments, cultures were harvested. Equal amounts of whole cell lysate proteins (20–50 µg/lane) were prepared and subjected to electrophoresis in 4–15% SDS polyacrylamide gradient gels [11]. Proteins were transferred to Immobilon-P membranes (Millipore, Billerica, MA), and membranes were probed sequentially with primary antibodies specific for Type I collagen (Southern Biotechnology, Birmingham, AL), Nab2 (IC4, C6), Egr-1 (C19), tubulin (Sigma-Aldrich, St. Louis, MO), actin (C2) (all from Santa Cruz Biotechnology, Santa Cruz, CA) or phospho-Smad2 (Ser^465,467^) and Smad2 (both from Cell Signaling, Beverly, MA). Membranes were then incubated with appropriate secondary antibodies and subjected to enhanced chemiluminescence detection using ECL Reagent (Amersham-Pharmacia, Piscataway, NJ).

### Immunoprecipitation- immunoblot analysist

Equal aliquots of whole cell lysates were immunoprecipitated using antibodies against Egr-1 (588, Santa Cruz). Immunoprecipitated proteins were subjected to Western analysis as above, using primary antibodies specific for HDAC1 (C19) or Nab2 (IC4) (both from Santa Cruz).

### Transient Transfection Assays

The reporter constructs 772COL1A2-CAT harboring the −772/+58 bp fragment of the human proα2(I) collagen gene [20], 376COL1A2-luc harboring [21] and pEBS_4_-luc harboring four copies of the minimal Egr-1 responsive element (EBS) linked to luciferase [22] were used for transient transfection assays. Subconfluent cultures of human foreskin or mouse NIH3T3 fibroblasts in serum-free media were transfected using Superfect reagent (Qiagen, Valencia, CA). Following 24 h incubation with TGF-β1, cultures were harvested, and whole cell lysates were prepared and assayed for their CAT or luciferase activities [23]. In each experiment, fibroblasts were cotransfected with Renilla luciferase pRL-TK plasmids (Promega) as a control for transfection efficiency. Experiments were performed in triplicates and repeated at least twice.

### Infection with adenovirus and siRNA knockdown experiments

Adenoviral recombinants containing wildtype Egr-1 (Ad-Egr-1), or a constitutively active mutant Egr-1 (Ad-mEgr-1), EGFP (Ad-EGFP), and NAB2 (Ad-Nab2) co-expressing the green fluorescent protein (GFP) [24] were amplified. Confluent human skin fibroblasts in serum-free media were infected with adenovirus. After 24h of incubation, fresh media with 10% FBS were added, and 24 h later whole cell lysates or total RNA were harvested for analysis.

To deplete cellular Nab2 skin fibroblasts were transfected with a Nab2-specific siRNA or an irrelevant negative control siRNA (Applied Biosystems/Ambion, Austin, TX) using Lipofectamine™ RNAiMAX (Invitrogen, Carlsbad, CA) transfection reagent following manufacture's protocol. Forty eight hour later, fibroblasts were incubated with TGF-β1 for a further 24 h. At the end of incubation cells were harvested, and whole cell lysates or total RNA were analysed. Knockdown efficiency was evaluated by determining the levels of Nab2 protein by Western analysis.

### Chromatin immunoprecipitation assays

Chromatin immunoprecipitation (ChIP) assays were performed using Ez-Magna CHIP ™ G (Millipore, Billerica, MA) as described [12]. Briefly, confluent cultures of fibroblasts transfected with Nab2 or empty vector were incubated with TGF-β1 (10 ng/ml) for 120 min or 24 h. Formaldehyde (final concentration 1%) was added to the cultures to cross-link chromatin. Nuclear extracts were then prepared and sonicated on ice to generate chromatin DNA fragments with an average length of 500–1000 bp. Aliquots of lysates were immunoprecipitated with antibodies to CHD3/4 (sc-11378), HDAC1 (C9), Nab2 (IC4) or Egr-1 (588) (all from Santa Cruz), or acetylated histone H4 (Upstate/Millipore, Billerica, MA). DNA was recovered, and PCR amplification of the captured sequences was performed using primers complementary to the COL1A2 promoter region harboring both of the putative Egr-1-binding sites [10]. The primer sequences were forward primer, 5′-CTACAGGGCACAGGTGAGG- 3′, and reverse primer, 5′-AAAGCCCGGATCTGCCCTA-3′, to generate a 422-bp amplification product. DNA samples were analyzed by electrophoresis in 2% agarose gels stained with ethidium bromide. Input samples were used as controls.

### Immunofluorescence microscopy

Foreskin fibroblasts seeded in 12-well plates (10^4^ cells/well) were transfected with Nab2 plasmids, followed by incubation in serum-free media with TGF-β1 for indicated periods. Cultures were then fixed and permeabilized with paraformaldehyde and 100% methanol, and incubated with antibodies to Nab2 (Santa Cruz) or to Type I collagen (Southern Biotechnology) at a 1∶100 dilution, followed by Alexa-fluor secondary antibodies (Invitrogen). Nuclei were identified using 4,6-diamidino-2-phenylindone (DAPI). Subcellular distribution of immunofluorescence was then evaluated using a Nikon Eclipse TE200 microscope (Fryer Company, IL). Acetylated histone H4 (Ac-H4) and HDAC1 expression was further examined by fluorescence confocal microscopy as above [11]. Cultures were incubated with anti rabbit human Ac-H4 (Upstate) or HDAC1 (Santa Cruz) antibodies at a 1∶100 dilution, followed by FITC-labeled secondary antibodies (Promega). For tissue immunofluorescence, the deparafinized sections were subjected to antigen retrieval followed by incubation with anti mouse αSMA (Sigma-Aldrich) at 1∶200 dilution for 1 hour at room temperature followed by Alexa-fluor-labeled secondary antibodies (Invitrogen). Slides were mounted and subcellular distribution of immunofluorescence was then evaluated under a Zeiss UV Meta 510 confocal microscope (Carl Zeiss Inc, Jena, Germany).

### Immunohistochemistry

Samples were obtained by skin biopsy from the affected forearm from six patients with scleroderma, and from the forearm of three healthy adults. The protocols for tissue collection were approved by the Institutional Review Boards for Human Studies at Northwestern University and Kanazawa University. Four µm thick paraffin-embedded skin sections were deparaffinized, rehydrated and immersed in TBS-T buffer (Tris-buffered saline- 0.1%Tween 20) followed by target retrieval solution (DAKO, Carpinteria, CA). Following incubation of the slides using primary antibodies against anti-mouse Nab2 monoclonal antibodies (Santa Cruz, 1∶50 dilution), anti-rabbit phospho-Smad2 (Cell Signaling Technology, 1∶100 dilution), and anti-rabbit Egr-1 (Santa Cruz, 1∶100 dilution), appropriate goat anti-mouse IgG or donkey anti goat IgG (Dako) were applied as secondary antibodies. Bound antibodies were detected using DAKO Envision + System according to the manufacturer's instructions. phospho-Smad2 (Cell Signaling Technology, Danvers, MA), After counterstaining with hematoxylin, sections were mounted with Permount (Fisher Scientific, Pittsburgh, PA) and viewed under Zeiss Axioskop Upright Microscope with CRi spectral CCD camera (Carl Zeiss Inc, Jena, Germany). Substitution of primary antibody with non-specific mouse IgG served as negative control.

Skin was also harvested from six month-old Nab2 null (Nab2^−/−^) mice and C57BL/6 isogenic controls (17). Four µm thick sections of paraffin-embedded skin tissue were stained with hematoxylin and eosin (H&E). To analyze the collagen content and architecture in the lesional skin, deparafinized sections were stained with Picrosirius red and viewed under polarized light [19].

### Statistical Analysis

Statistical significance was determined using the unpaired Student's t-test. A p value <0.05 was considered significant.

## Results

### TGF-β stimulates Nab2 expression in normal skin and lung fibroblasts

**Figure 1:**
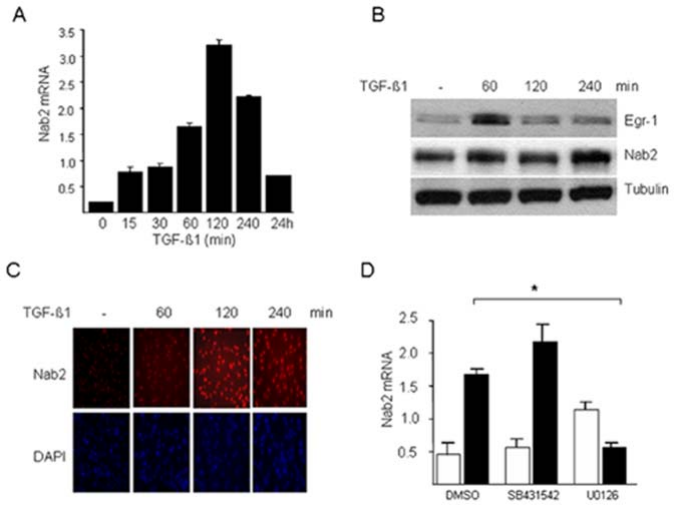
TGF-β stimulates Nab2 expression. Confluent cultures of human foreskin fibroblasts were incubated with TGF-β1 (10 ng/ml) for indicated periods. A, D Total RNA was isolated and analysed by real-time quantitative PCR. Results, normalized with actin, are the means±S.D. of triplicate determinations from a representative experiment. B. Whole cell lysates were subjected to Western analysis. Representative immunoblots. C. Fibroblasts were fixed and stained with anti-Nab2 antibodies, or DAPI to detect nuclei, and viewed by immunofluorescence microscopy (original magnification ×100).

We have shown previously that TGF-β stimulated the expression of Egr-1 in normal fibroblasts [10], [11]. Furthermore, ectopic expression of Egr-1 was sufficient to stimulate collagen synthesis in these cells. These finding suggested a novel role for Egr-1 in mediating profibrotic TGF-β responses. We hypothesized that as an endogenous cofactor for Egr-1, Nab2 might be involved in modulating TGF-β responses. To begin to explore the function of Nab2 in fibroblasts biology and TGF-β signaling, we first examined the regulation of Nab2 by TGF-β. Compared to the rapid and transient increase in Egr-1 mRNA expression that was induced by TGF-β, Nab2 showed a comparatively delayed stimulatory response that persisted even after 24 h in normal skin and lung fibroblasts (Fig. 1 and data not shown). Western analysis demonstrated rapid accumulation of Egr-1 protein that peaked at 60 min of TGF-β1 incubation, whereas the Nab2 response in the same cells peaked later at 240 min. Immunofluorescence studies showed a time-dependent progressive increase in Nab2 within the nucleus, indicating that enhanced synthesis of Nab2 was accompanied by its nuclear translocation in TGF-β1-treated fibroblasts (Fig. 1C). The stimulation of Nab2 expression by TGF-β1 was completely abolished by pretreatment of the cultures with the MEK1 inhibitor U0126, whereas the ALK5 inhibitor SB431542 did not significantly affect the response (Fig. 1D).

### Nab2 blocks Egr-1-dependent transcriptional activity

To examine the modulation of Egr-1-dependent responses by Nab2 in the context of fibrogenesis, confluent NIH3T3 fibroblasts were transfected with adenovirus for Egr-1 (Ad-Egr-1), or a mutant Egr-1 (Ad-Egr-1m) that is resistant to Nab2 due to a mutation in the Nab2-binding repression domain [24]. The fibroblasts were then cotransfected with pEBS_4_-luc, a synthetic promoter-luciferase reporter construct containing four canonical Egr-1 binding sites, and incubated for an additional 24 h. The results of transient transfection assays showed that ectopic Nab2 completely blocked the stimulation of pEBS_4_-luc activity induced by wildtype Egr-1, as shown previously [25], whereas stimulation induced by the Nab2-resistant mutant Egr-1 was unaffected (data not shown).

### Nab2 abrogates TGF-β-induced stimulation of Type I collagen synthesis and myofibroblast differentiation

**Figure 2:**
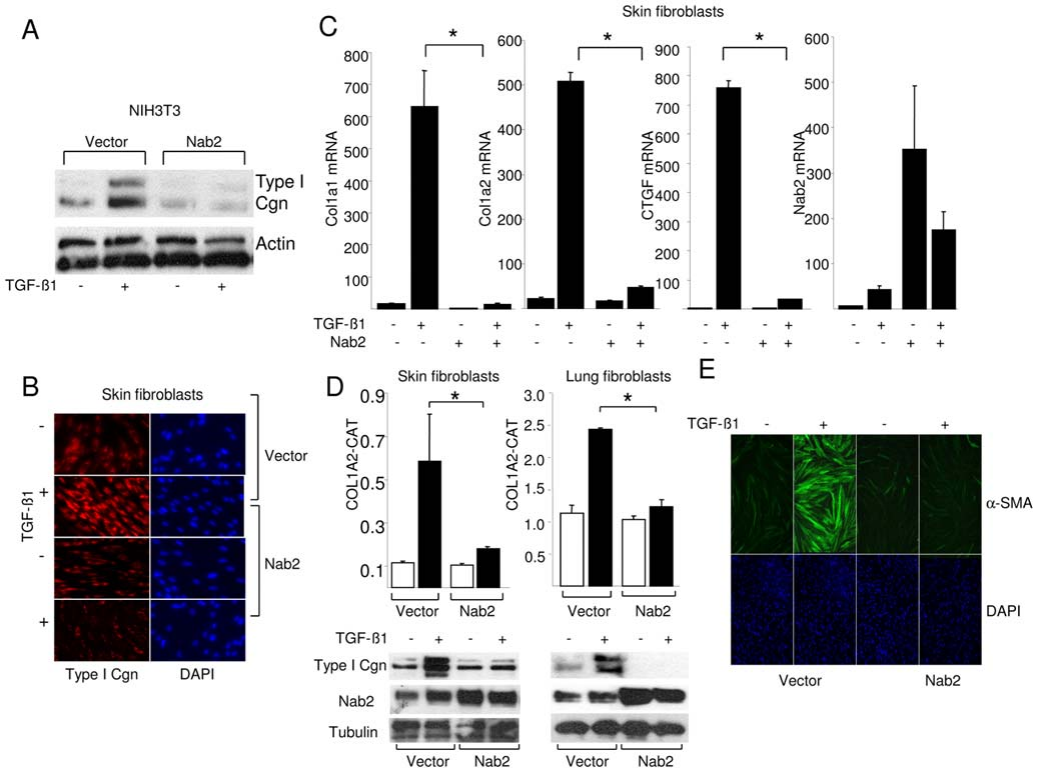
Nab2 abrogates TGF-β responses. Cultures of fibroblasts were cotransfected with Nab2 expression vectors or empty vector and harvested following incubation with TGF-β1 for 24 or 48 h. A. Whole cell lysates were examined by Western analysis. Representative immunoblots. B. Fibroblasts were fixed, incubated with antibodies to Type I collagen, and examined by immunofluorescence microscopy (original magnification ×400). Nuclei were identified by DAPI (blue). C. Total RNA was isolated and examined by real-time qPCR. Results, normalized with actin, are the means±S.D. of triplicate determinations from a representative experiment. D. Fibroblasts were cotransfected with 772COL1A2-CAT. Cell lysates were assayed for their CAT activities (upper panels) or examined by Western analysis (lower panels). Results of CAT assays, normalized with Renilla luciferase, are expressed as means±S.D. of triplicate determinations from a representative experiment. Open boxes, untreated fibroblasts; closed boxes, TGF-β-treated fibroblasts. *p<0.005. E. After 48 h fibroblasts were fixed, incubated with antibodies to α-SMA (green) and examined by immunofluorescence microscopy. Nuclei were identified by DAPI (blue). Representative images (original magnification ×100).

We had suggested previously that endogenous Egr-1 plays an important role in mediating TGF-β responses [10], prompting us to consider therefore whether Nab2 might function as a negative regulator of TGF-β signaling. To investigate the modulation of TGF-β-induced transcriptional activation, fibroblasts were transfected with Nab2, and incubated in the presence and absence of TGF-β. The results showed that ectopic Nab2 abrogated TGF-β-induced stimulation of collagen synthesis in mouse (Fig. 2A) and human fibroblasts (Fig. 2B). In addition, stimulation of Egr-1-dependent transcription (pEBS_4_-luc) by TGF-β was also abrogated (data not shown). Moreover, ectopic Nab2 also abrogated TGF-β-induced stimulation of Type I collagen synthesis, COL1A2 promoter activity, and COL1A1and COL1A2 mRNA expression in human skin and lung fibroblasts (Figs. 2C and D).

**Figure 3:**
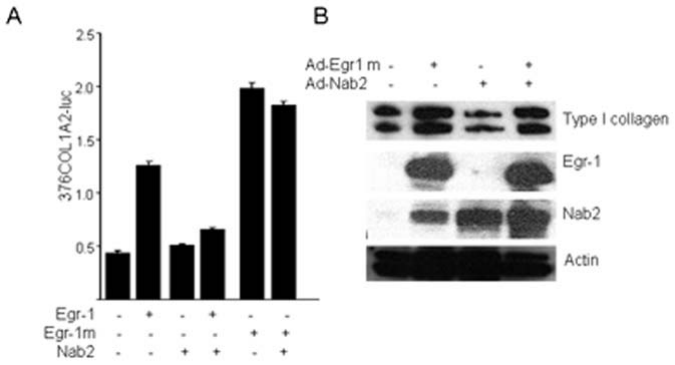
Nab2 blocks Egr-1-dependent collagen stimulation. A. Foreskin fibroblasts were cotransfected with expression vector for wildtype or mutant Egr-1 along with Nab2 or empty vector, and 376COL1A2-luc_reporter constructs. Following incubation of cultures for 24 h, cell lysates were prepared and assayed for their luciferase activities. Results, normalized with Renilla luciferase, are expressed as means±S.D. of triplicate determinations. *p<0.005. B. Foreskin fibroblasts were infected with Ad-Egr-1m along with Ad-Nab2. Following 24 h incubation, whole cell lysates were prepared and subjected to Western analysis. Representative immunoblots.

Myofibroblasts play a central role in the development and progression of tissue fibrosis. Because TGF-β stimulates myofibroblasts differentiation, and as myofibroblasts accumulation was found to be attenuated in Egr-1 null mice [26]. we investigated the regulation of myofibroblast differentiation by Nab2. For this purpose, fibroblasts were transfected with Nab2, and following incubation with TGF-β for 48 h, examined by immunofluorescence. The results showed that whereas TGF-β induced a marked increase in α-SMA expression and stress fiber incorporation in transfected fibroblasts, ectopic Nab2 abolished this stimulatory response (Fig. 2E). Induction of Smad2 phosphorylation, a specific marker for canonical TGF-β signaling, was unaffected by ectopic Nab2 (data not shown). Together, these results demonstrate that Nab2 has potent inhibitory effect on TGF-β-driven stimulation of fibrotic responses independent of canonical Smad signaling. We speculated that these inhibitory effects of Nab2 involved repression of TGF-β-induced Egr-1 signaling. To explore this possibility, additional transient transfection assays were performed. The results showed that ectopic expression of Nab2 blocked the stimulation of COL1A2-luc activity and Type I collagen synthesis induced by Egr-1 (Figs. 3A and B, and data not shown), whereas stimulation induced by the Nab2-resistant mutant Egr-1 could not be prevented by Nab2. Taken together, these results not only confirm that the inhibitory effect of Nab2 was selective for Egr-1-dependent transcriptional responses, but also serve to further underline the essential role of endogenous Egr-1 in mediating the stimulation of collagen gene expression and myofibroblasts differentiation induced by TGF-β.

**Figure 4:**
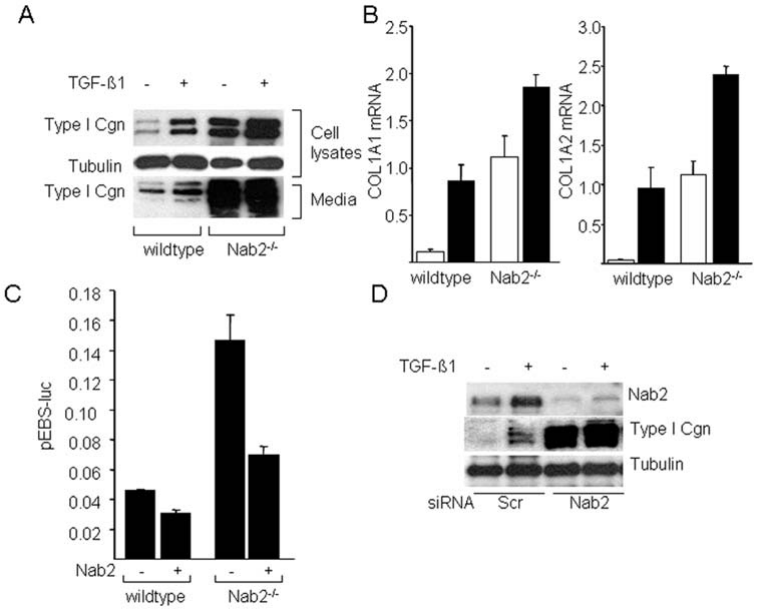
Enhanced collagen stimulation in Nab2-deficient fibroblasts. Wildtype and Nab2^−/−^ mouse embryonic fibroblasts (MEFs) cultured in parallel were incubated with or without TGF-β1 for 24 h. A. Whole cell lysates and culture supernatants were subjected to Western analysis. Representative immunoblots. B. Total RNA was subjected to real-time qPCR analysis. Results, expressed relative to 18 s, are the means±S.D. of triplicate determinations from a representative experiment. Open boxes, untreated fibroblasts; closed boxes, TGF-β-treated fibroblasts. *p<0.005. C. MEFs were transfected with pEBS_4_-luc inpresence or absence of Nab2. Following 24 h incubation, cultures were harvested and cell lysates were assayed for their luciferase activities. The results, normalized with Renilla luciferase, are the means±S.D. of triplicate determinations. D. siRNA knockdown. Normal dermal fibroblasts were transfected with Nab2 siRNA or irrelevant negative control siRNA, and incubated with TGF-β. Twenty-four h later, fibroblasts were harvested. Levels of Nab2 and collagen were determined by Western analysis of whole cell lysates.

To investigate the role that endogenous Nab2 might play in the physiologic regulation of collagen gene expression, two complementary approaches were taken. First, mouse embryonic fibroblasts (MEFs) were generated from Nab2-null mice and studied in vitro. Whereas Nab2^−/−^ MEFs in vitro displayed morphology and proliferation rates indistinguishable from wildtype MEFs under standard culture conditions (data not shown), marked alterations in their biosynthetic phenotypes were evident. Western analysis and real-time qPCR revealed substantial increases in the constitutive rate of collagen synthesis, and in COL1A1 and COL1A2 mRNA expression in Nab2^−/−^ MEFs compared to wildtype MEFs cultured in parallel (Figs. 4A and B). Furthermore, incubation of Nab2^−/−^ MEFs with TGF-β resulted in further enhancement of collagen gene expression. A potential mechanistic explanation to account for the enhanced collagen gene transcription seen in Nab2^−/−^ MEFs was provided by the finding that the basal activity of the Egr-1-responsive reporter pEBS_4_-luc was constitutively elevated in Nab2^−/−^ MEFs, and could be partially normalized by rescuing these cells with ectopic Nab2 (Fig. 4C). These results suggest the basal Egr-1 activity is unopposed in Nab2^−/−^ MEFs. Next, we used an siRNA knockdown approach to reduce cellular Nab2 expression. Transfection of normal dermal fibroblasts with a Nab2 siRNA reduced the baseline levels of Nab2, and abrogated the stimulation of Nab2 expression by TGF-β (Fig. 4D). Transfection of an irrelevant negative control siRNA (lanes 1,2) had no effect on Nab2 expression. The inhibitory effect of the siRNA was specific for Nab2, since levels of tubulin were unaffected. Down-regulation of cellular Nab2 was accompanied by significantly enhanced stimulation of collagen gene expression in response to TGF-β (Fig. 4D, right panel). Together, the results of these complementary genetic and siRNA-mediated loss-of-function experiments point to an important physiologic function of cellular Nab2 in negative modulation of basal and TGF-β-induced collagen gene expression.

### Nab2 negatively regulates dermal collagen accumulation in vivo

**Figure 5:**
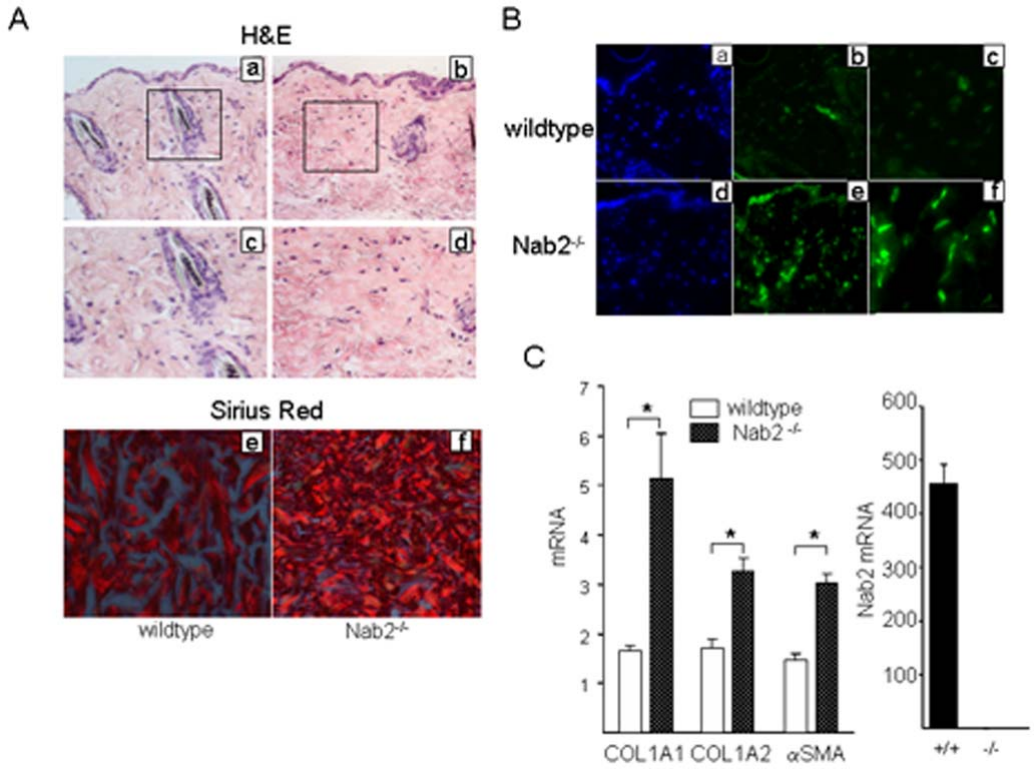
Increased skin collagen accumulation in Nab2 ^−/−^ mouse. Skin from Nab2^−/−^ mice and wildtype mice was harvested. A. Tissues were stained with hematoxylin and eosin (a–d) or picrosirius red (e,f). Original magnification ×100 (a, b), ×400 (c–f). B. Immunofluorescence using antibodies to α-smooth muscle actin (green). Nuclei were identified by DAPI (blue). Original magnification ×100 (a, d), ×200 (b, c, e, f). C. Total RNA was harvested and was subjected to real-time qPCR analysis. Results, expressed relative to 18S, are the means±S.D. of triplicate determinations from a representative experiment.

To further investigate the role of Nab2 in the regulation of extracellular matrix homeostasis, skin from six-months old Nab2^−/−^ mice and wildtype littermates was harvested. Mice deficient in Nab2 were produced at the expected mendelian ratios and showed no overt phenotype at this age [17]. Histological examination of skin biopsies stained with hematoxylin and eosin showed that Nab2^−/−^ mice had a more compact and sclerotic dermis than wildtype controls (Fig. 5A, a–d). The striking epidermal hyperkeratinization previously described in mice lacking both Nab1 and Nab2 (17) was not seen. To evaluate the collagen content and architecture of the skin, deparafinized sections were stained with Picrosirius red and viewed under polarized light. Increased dermal accumulation of strongly red birefringent collagen fibers was noted in Nab2^−/−^ mice compared to wildtype littermates (Fig. 5A, panels e,f). Immunofluorescence showed increased numbers of α-smooth muscle actin-positive myofibroblasts in the dermis from Nab2^−/−^ mice (Fig. 5B, compare panels c and f). Furthermore, mRNA levels for COL1A1, COL1A2 and α-smooth muscle actin were also significantly increased in the skin of Nab2^−/−^ mice (Fig. 5C).

### Nab2 recruits NuRD complex to the COL1A2 promoter and attenuates histone H4-hyperacetylation

**Figure 6:**
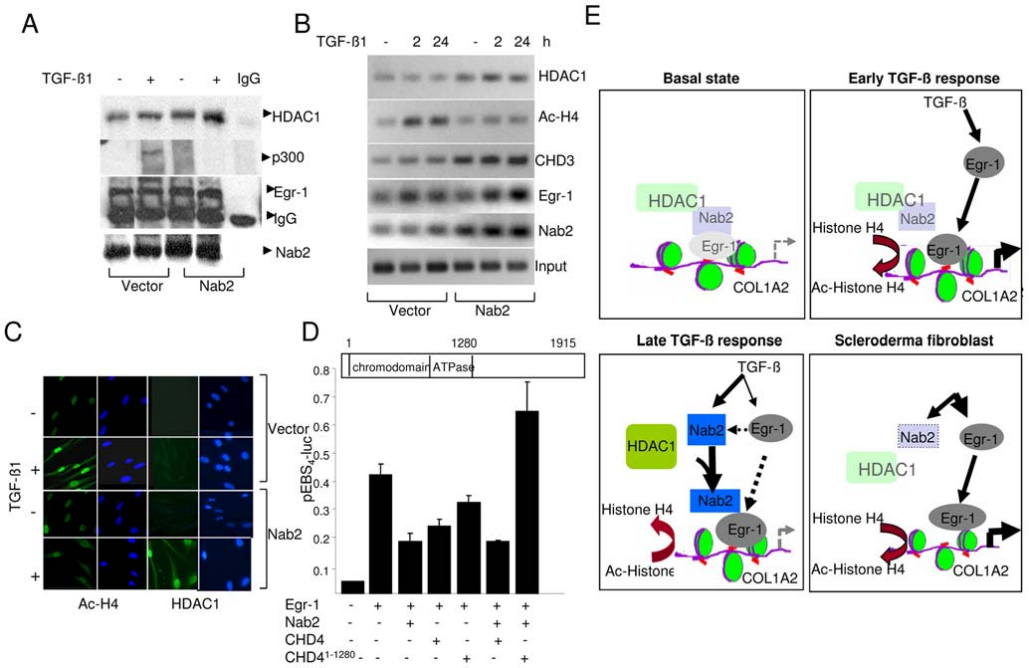
Ectopic Nab2 recruits NuRD complex to the COL1A2 promoter. A. Foreskin fibroblasts transfected with Nab2 were incubated with TGF-β1 for 24 h. Whole cell lysates were immunoprecipitated with antibodies against Egr-1 and immunoblotted using indicated antibodies. Representative immunoblots. B. ChIP assays. Fibroblasts transfected with Nab2 were formaldehyde cross-linked and immunoprecipitated with indicated antibodies, followed by amplification of the captured DNA using COL1A2-specific primers. Input genomic DNA was used as positive control. C. Fibroblasts transfected with Nab2 were incubated with TGF-β for 2 h. Cells were then fixed, incubated with indicated antibodies and examined by immunofluorescence confocal microscopy. Nuclei were identified by DAPI (blue). Representative images. Original magnification ×400. D. NIH3T3 fibroblasts were cotransfected with expression vectors for Egr-1, Nab2, wildtype CHD4 or mutant CHD4 del(1–1280) or empty vector, along with pEBS_4_-luc. Cultures were harvested 24 h later, and cell lysates were assayed for their luciferase activities. The results, normalized with Renilla luciferase, are the means±S.D. of triplicate determinations. *p<0.005. E. Cartoon illustrating the mechanism underlying modulation of collagen gene expression by Nab2. In unstimulated fibroblasts (basal state), small amounts of Egr-1 and Nab2 are constitutively associated with the COL1A2 promoter. Upon transient TGF-β stimulation, Egr-1 is induced and recruited to the COL1A2 promoter, where together with p300 it enhances histone H4 hyperacetylation and stimulates transcription. More sustained TGF-β stimulation leads to increased Nab2 expression and its accumulation in the Egr-1-COL1A2 transcriptional complex, with HDAC1 recruitment, H4 deacetylation, and transcriptional repression. In scleroderma fibroblasts, defective Nab2 induction or function might results in unopposed Egr-1 signaling and target gene transcription. For full explanation, see text (Discussion).

Previous studies showed that Nab2 can interact with the nucleosomal remodeling and deacetylation (NuRD) complex that comprises histone deacetylase HDAC1/2 and CHD4 [27], [28], [29], [30], [31], [32]. To begin to explore the mechanistic basis for the repression of TGF-β responses in the presence of ectopic Nab2, cell lysates were prepared from fibroblasts harboring ectopic Nab2 that had been exposed to TGF-β. The lysates were immunoprecipitated with antibodies to Egr-1, and immunoblotted with antibodies to HDAC1. The results showed low levels of constitutive interaction between cellular Egr-1 and HDAC1 in unstimulated fibroblasts (Fig. 6A). The interaction was considerably enhanced in Nab2-transfected fibroblasts in the absence, and even more so in the presence, of exogenous TGF-β. Western analysis of the same whole cell lysates showed that ectopic Nab2 completely abrogated TGF-β-induced stimulation of Type I collagen synthesis, as expected (data not shown).

These results suggested that ectopic overexpression of Nab2 exerted a stimulatory effect enhancing the interaction of cellular Egr-1 with HDAC1. To investigate the role of Egr-1 in the abrogation of TGF-β-induced fibroblast responses by ectopic Nab2, chromatin immunoprecipitation (ChIP) assays were performed. For this purpose, fibroblasts expressing ectopic Nab2 were incubated with TGF-β for indicated periods. Following formaldehyde cross-linking, chromatin was isolated and subjected to ChIP and amplification of captured DNA sequences. Whereas only low amounts of COL1A2 promoter associated with HDAC1could be detected in fibroblasts harboring empty vector, ectopic Nab2 expression was accompanied by increased accumulation HDAC1, as well as Nab2, on the Egr-1-binding region of COL1A2 promoter fragments (Fig. 6B). Accumulation of CHD4, another member of the NuRD complex, was also enhanced by TGF-β, and was markedly elevated in Nab2-transfected fibroblasts. In contrast, TGF-β-induced histone H4 hyperacetylation at the COL1A2 locus was markedly attenuated in the presence of ectopic Nab2. Increased nuclear levels of HDAC1 in fibroblasts harboring ectopic Nab2, and attenuation of histone H4 hyperacetylation, were confirmed by immunofluorescence confocal imaging (Fig. 6C). Together, these results suggest that ectopic Nab2 expression in fibroblasts stimulated the recruitment of HDAC1 and CHD4, with ensuing decrease in histone hyperacetylation at the COL1A2 promoter.

Based on these results, we surmised that NuRD complex recruitment to the COL1A2 promoter may play a role in repression of the stimulation of collagen gene expression in the presence of ectopic Nab2. This notion was explored by disrupting NuRD function. For this purpose, transient transfection assays were performed in the presence of wildtype CHD4 or CHD4del^(1–1280)^, a dominant negative CHD4 mutant that binds to Nab2 but lacks the ATPase domain required for nucleosome remodeling [27]. The results of transfection assays showed that ectopic Nab2 prevented the stimulation of Egr-1-dependent transcription in presence of wildtype CHD4, as expected, but it failed to block the response in fibroblasts harboring the mutant CHD4, suggesting a critical role of the NuRD remodeling complex in Nab2-mediated repression (Fig. 6D). The speculative model for the proposed mechanism of action is illustrated schematically in Fig. 6E.

### Elevated Nab2 expression in scleroderma skin biopsies

**Figure 7:**
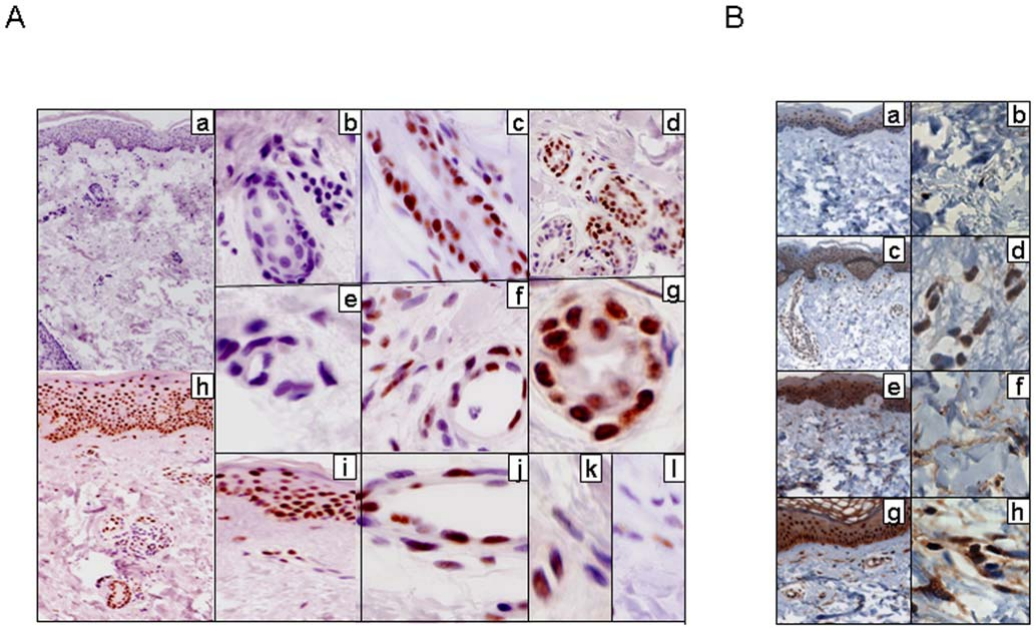
Elevated Nab2 expression in scleroderma skin biopsies. Lesional skin biopsies were obtained from patients with scleroderma (n = 6) and healthy controls (n = 3), and processed for immunohistochemistry as described under Methods. A. Nab2 expression. Skin biopsies from healthy controls (a, b, e) and scleroderma patients (c, d, f, g, h–l). Original magnification ×100(a, h), ×400 (b, d, i), ×630 (c, e, f, j, k, l). Note uniformly intense Nab2 nuclear immunoreactivity in epithelial cells in scleroderma skin biopsies (h, i, l), as well as in perifollicular and periglandular (c, d, g) epithelial cells and vascular endothelial cells (f, j), compared to weak and variable Nab2 expression seen in occasional dermal fibroblasts (k, l). Representative photomicrographs; similar immunostaining pattern was observed in all six scleroderma skin biopsies. B. Phospho-Smad2 (a–d) or Egr-1 (e–h) expression. Nuclei are counterstained with hematoxylin (blue). Note strong immunostaining in dermal fibroblasts in scleroderma (d, h) skin biopsies compared to healthy controls (b, f).

We showed previously that aberrant TGF-β signaling in scleroderma was associated with sustained up-regulation of Egr-1 expression in the lesional skin [11]. The present results revealed a potentially important functional role for Nab2 in the regulation of Egr-1 activity. To examine the expression of Nab2 in scleroderma, biopsies of lesional skin from patients with scleroderma and dorsal forearm biopsies from healthy controls were studied in parallel. The clinical features of the patients are shown in Table 2. The results of immunohistochemistry showed virtual absence of Nab2 healthy skin. In marked contrast, very strong and uniform Nab2 immunostaining was evident in each scleroderma skin biopsy (Fig. 7). In these samples, Nab2 accumulation was nuclear and primarily localized to the epidermis. In addition to keratinocytes, dermal blood vessels, hair follicles, eccrine glands and occasional infiltrating round cells also showed strong immunostaining. In contrast, however, only occasional fibroblastic cells were positive for Nab2. These were distributed throughout the dermis and showed faint and inconsistent staining. Immunostaining was specific for Nab2, as substitution of the primary antibody with nonspecific mouse IgG resulted in the absence of staining (data not shown). In the same skin biopsies, Egr-1 immunostaining was evident in majority of dermal fibroblasts. Furthermore, most dermal fibroblasts showed strong expression of nuclear phospho-Smad2, indicating active TGF-β signaling in these cells.

**Table 2:** Clinical characteristics of scleroderma patients studied.

Skin biopsy samples	Age (years)	Sex	Disease duration (years)	Modified Rodnan skin score (1–51)
Scleroderma 1	43	M	0.1	24
Scleroderma 2	29	F	2.2	29
Scleroderma 3	54	F	11.0	4
Scleroderma 4	64	F	20.0	2
Scleroderma 5	63	M	13.0	7
Scleroderma 6	43	F	0.6	24

M, male; F, female.

## Discussion

We recently identified Egr-1 as a novel intracellular mediator of TGF-β responses [10]. The expression of Egr-1 was markedly up-regulated in the fibrotic lesion in a mouse model of scleroderma, as well as in skin and lung biopsies from patients with scleroderma [11]. The expression and activity of Egr-1 are normally tightly regulated through a variety of mechanisms. The present studies focused on Nab2, a non-DNA-binding cofactor for Egr-1. The results demonstrated that Nab2 expression was induced by TGF-β, and ectopic Nab2 abrogated TGF-β-dependent fibrotic responses such as stimulation of collagen gene expression and myofibroblasts differentiation, by blocking Egr-1-dependent target gene transcription. Mice lacking Nab2 showed evidence of enhanced fibrogenesis in the skin, and explanted Nab2-null fibroblasts showed constitutive Egr-1 transcriptional activity and elevated collagen gene expression in vitro. Furthermore, repression of endogenous Nab2 using siRNA knockdown was accompanied by enhancement of Egr-1-dependent TGF-β responses in normal fibroblasts. Nab2 promoted HDAC1 recruitment to the COL1A2 promoter and abrogated histone hyperacetylation induced by TGF-β. While Nab2 expression was dramatically up-regulated in epithelial cells in scleroderma skin biopsies, consistent with activated TGF-β signaling in the fibrotic milieu, dermal fibroblasts from biopsies that were strongly immunopositive for Egr-1 and phospho-Smad2 showed a paucity of Nab2 expression. These findings indicate that in the context of TGF-β-induced profibrotic responses, Nab2 functions as an important negative regulator, and impaired Nab2 expression or function in activated dermal fibroblasts might contribute to the persistence or progression of skin fibrosis in scleroderma.

The expression of Egr-1 during acute injury is induced by serum, platelet-derived growth factor (PDGF), TGF-β, hypoxia and oxidative stress, and in turn, Egr-1 stimulates the expression of PDGF, tissue factor and TGF-β itself [9], [13], [14], [33], [34]. Egr-1 is strongly expressed in wounded skin, highlighting its critical role in normal wound healing and tissue repair [26]. Like other transcription factor, Egr-1 interacts with cofactors that can modulate the transcription of Egr-1 target genes. Nab2, originally identified by yeast two-hybrid assays, is a non-DNA binding cofactor that directly interacts with a conserved 34-amino acid sequence in the R1 domain of Egr-1. Deletion of the R1 domain resulted in increased Egr-1 activity [13], [14], [35]. We show here that TGF-β stimulated the expression of Nab2 in normal fibroblasts. This response was delayed compared to Egr-1, and was abolished by an inhibitor of MEK1, indicating that it was mediated via a MAP kinase signaling pathway.

The in vivo functions of Nab2 are incompletely understood. In vascular cells, Nab2 repressed the Egr-1-mediated stimulation of PDGF, TGF-β, tissue factor and PPAR-γ transcription [34], [36], [37], [38], [39], [40], and inhibited angiogenic responses in vivo [41], [42]. However, it has been shown that Nab2 can also serve to positively modulate Egr-1-dependent transcription, for example enhancing rather than repressing the Egr-1-induced stimulation of Fas ligand expression [21]. The present studies reveal a novel physiological function for Nab2 in the regulation of fibrogenesis. While loss of fibroblast Nab2 was accompanied by constitutive Egr-1 activity that could be partially rescued by ectopic Nab2, in addition to elevated collagen gene expression and enhanced TGF-β responses, ectopic Nab2 abrogated the stimulation of fibrotic responses such as collagen production and myofibroblast differentiation induced by TGF-β. In previous studies, mice deleted for Nab2 appeared to have no obvious phenotype, whereas mice lacking both Nab1 and Nab2 developed peripheral neuropathy and abnormalities in skin development, and suffered from early lethality [17], [43], [44]. While these observations suggest functional redundancy between Nab1 and Nab2, we found here that Nab2 null mice showed excessive collagen accumulation in the dermis, and explanted Nab2-null fibroblasts in vitro showed both constitutively elevated Egr-1 transcriptional activity that could be normalized with ectopic Nab2, and elevated collagen synthesis, suggesting an important biological role for Nab2 in regulating Egr-1-dependent fibrogenesis.

It is worth noting that not all TGF-β-induced responses are abrogated by Nab2. Indeed, transcriptional profiling analysis using DNA microarrays indicated that the expression of multiple TGF-β- target genes such as PLOD2 and SMAD7, and few more in TGF-β-stimulated skin fibroblasts could not be inhibited by ectopic Nab2 (Bhattacahryya S et al., Ms. in preparation). A previous study showed that for selected Egr-1 target genes Nab2 was capable of serving as a coactivator rather than repressor for Egr-1-dependent transcription [21].

Nab2 interacts with members of the NuRD histone deacetylase complex [27], [28], [29], [30], [31], [32]. The present results indicate that repression of TGF-β responses by ectopic Nab2 was associated with recruitment of both HDAC1 and CHD4 to the COL1A2 promoter, and reduced local histone H4 acetylation. Furthermore, dominant negative mutant CHD4 abrogated the inhibitory effect of ectopic Nab2 on Egr-1 activity. We showed previously that TGF-β induced the recruitment of p300 to the COL1A2 promoter, resulting in histone H4 hyperacetylation [45]. Based on the present results, we hypothesize that ectopic expression of Nab2 might interfere with p300 function or H4 histone acetylation at the COL1A2 locus by recruiting HDAC1, thereby inhibiting COL1A2 transcription. Sustained TGF-β stimulation might result in a relative imbalance between Nab2 and Egr-1 favoring the former, resulting in inhibition of selected Egr-1-dependent genes. This notion is illustrated schematically in Fig. 7.

We have shown that Egr-1 expression was up-regulated in scleroderma skin biopsies [11]. Strong Egr-1 staining was localized primarily to fibroblastic cells within the reticular dermis, presumably reflecting activity of TGF-β in the fibrotic cellular milieu. The present results show that while Nab2 expression was highly elevated in scleroderma skin biopsies, in contrast to Egr-1, it was localized almost exclusively to the epidermis and epithelial cells lining dermal appendages, whereas dermal fibroblasts showed scant Nab2 expression.

In summary, we furnish novel insight into the pathogenesis of fibrogenesis by showing that the Egr-1 cofactor Nab2 is potently induced by TGF-β in normal fibroblasts, and it acts in a negative feedback loop for repressing Egr-1-dependent TGF-β signaling. We found a marked overexpression of Nab2 in scleroderma skin biopsies, where Nab2 was localized to epithelial cells, with only scant expression in fibroblastic cells in the dermis. These findings suggest that excessive TGF-β activity in the lesional skin results in Nab2 up-regulation in the epidermis, whereas in dermal fibroblasts, the primary effector cells responsible for skin fibrosis, a relative imbalance between Egr-1 and Nab2 expression exists. The results suggest that Nab2 forms part of a transcriptional circuitry that normally plays a negative feedback role by extinguishing acute Egr-1 activity induced by TGF-β, as well as hypoxia and other stimuli associated with injury and fibrogenesis. Since inducible Nab2 expression normally appears to be responsible for restoring basal homeostasis and fibroblasts quiescence, defective function of the Egr-1/Nab2 self-regulating activity might play an important role in the pathogenesis of fibrosis.
